# Subdistrict-Level Reproductive Number for Foot and Mouth Disease in Cattle in Northern Thailand

**DOI:** 10.3389/fvets.2021.757132

**Published:** 2021-11-10

**Authors:** Orapun Arjkumpa, Catalina Picasso-Risso, Andres Perez, Veerasak Punyapornwithaya

**Affiliations:** ^1^Animal Health Section, The 4th Regional Livestock Office, Department of Livestock Development, Khon Kaen, Thailand; ^2^Department of Veterinary Population Medicine, University of Minnesota, St. Paul, MN, United States; ^3^Faculty of Veterinary Medicine, Veterinary Public Health Centre for the Asia Pacific, Chiang Mai University, Chiang Mai, Thailand; ^4^Faculty of Veterinary Medicine, Center of Excellence in Veterinary Public Health, Chiang Mai University, Chiang Mai, Thailand

**Keywords:** subdistrict reproductive number, foot and mouth disease, serotype O, cattle, northern Thailand

## Abstract

Foot and mouth disease (FMD) is an important contagious transboundary disease that causes a significant economic loss for several countries. The FMD virus (FMDV) can spread very rapidly by direct and indirect transmission among susceptible animals. The complexity and magnitude of FMDV transmission at the initial stages of the epidemic can be expressed by the basic reproductive number (*R*_0_), and furthermore, control strategies can be assessed by the estimation of the effective reproductive number. In this study, we aimed to describe FMD outbreaks among smallholder cattle farms by subdistricts in the northern Thailand and compute the effective reproductive number for outbreaks caused by FMDV serotype O and overall serotypes, including serotype O, serotype A, and unidentified serotype, at the subdistrict level (*R*_*sd*_) using an epidemic doubling time method. Field data of FMD outbreaks during 2015–2017 that affected 94 subdistricts in northern Thailand were assessed to estimate the *R*_*sd*_. Results showed that 63.38% (90/142) of the FMD outbreak episodes in cattle were caused by FMDV serotype O. The average doubling time and the *R*_*sd*_ estimated of the outbreaks caused by FMDV serotype O and overall serotype were 2.80 and 4.67 months, and 1.06 and 1.04, respectively. Our results indicated that transmission of FMD in cattle at the subdistrict level in northern Thailand was not controlled (*R*_*sd*_ > 1), which indicates the endemicity of the disease in the region. Although control measures are in place, the results from this study highlighted the need for enhancing FMD monitoring and control strategies in northern Thailand.

## Introduction

Foot and mouth disease (FMD) is the foremost viral transboundary and highly contagious vesicular animal disease affecting cloven-hoofed animals (1, 2). FMD has an important impact on animal health and productivity and represents a threat to national, regional, and international trades globally (3). The disease is caused by the FMD virus (FMDV), a single-stranded positive-sense RNA virus belonging to the genus *Aphthovirus* of the Picornaviridae family (4, 5). Seven distinct non-cross-immunity FMDV serotypes are circulating globally, namely, A, O, C, Asia-1, South African territories 1, 2, and 3 (3, 5–7). The FMD morbidity rate in a completely susceptible livestock population can reach 100%, whereas the mortality rate tends to be low in adult animals (1–5%), reaching 20% in young categories (2). Different serotypes of FMDV were reported in 77% of the worldwide livestock population located in countries of Africa, the Middle East, and Asia and constrained zones of South America (7). In Southeast Asia, four FMDV serotypes including A, O, C, and Asia 1 have circulated over recent years (8). The incidence and serotype specificity of FMDV vary across different Asian countries (8, 9). During the past decade in Southeast Asia, FMDV serotype O outbreaks have been spreading and becoming endemic in certain areas, including Thailand and neighboring countries (8).

Thailand in Southeast Asia has been an FMD endemic country for more than 60 years except for a free zone located in the eastern region of the country (10). The most common circulation serotypes currently are O and A, predominantly reported in cattle herds (11, 12), and cases are aggregated in Chiang Mai province located in the northern region (13). In Thailand, agricultural and animal production potential is strong, with a cattle population of ~5,000,000 heads (14, 15). More than 30% of this cattle production occurs in the northern region, bordering Laos and Myanmar, with some exceptions of semi-intensive livestock farming. The extensive nature of cattle production increases the potential for transboundary FMD transmission and spread in the region representing a challenge to the FMD control in this area (16).

The understanding of FMD transmission dynamics in cattle populations has proven to be useful at the time to control outbreaks and epidemics (17–21). The estimation of the basic reproductive number (*R*_0_) is an essential first step toward disease control and surveillance to understand pathogen dissemination (22). The average number of secondary infections caused by a primary case in a completely susceptible population is commonly used to characterize the transmissibility potential of a disease in a population (22). This parameter is computed by the integration of components such as transmission route, the duration of the infectiousness, and the effective contacts among individuals in the population (22–26). As the epidemic progresses, the initial susceptible population starts to gain immunity having an impact on the reproductive number, depending on the time elapsed. Furthermore, the reproductive number can serve as a tool to quantify the effectiveness of control strategies used. If the effective reproductive number reaches values lower than one, this implies that every diseased individual will infect <1 other individual in the population, resulting in controlling disease spread (17).

The urgency associated with the control of FMD outbreaks in the field tends to impair the optimal collection of detailed and granular data to precisely estimate FMD transmission parameters. In Thailand, data at the subdistrict level are compiled and recorded; however, individual farm records tend to be missed (27). Nevertheless, different methods have been described to obtain robust estimates of FMD transmissibility, involving the nearest infectious neighbor, the susceptible and infectious modeling, and the epidemic doubling time method in which aggregated data can be analyzed (21, 25, 28). In Thailand, to the best of our knowledge, there was no report on the estimation of basic or effective reproductive number for FMD outbreaks in cattle, resulting in a gap in the knowledge of FMD transmissibility that limits the information needed for its control. Therefore, the present study aimed to estimate the subdistrict-level effective reproductive number for the 2015–2017 FMD epidemic among cattle farms in northern Thailand and to understand dynamic patterns of the disease, with the ultimate goal of informing decisions on FMD control in northern Thailand.

## Materials and Methods

### Data Source and Case Definition

In this study, data from passive (reported by the farmers) and active (subsequent official veterinary sampling using a cross-sectional study design) surveillance for FMD outbreak detection in cattle in the northern Thailand occurring between 2015 and 2017 from the National Animal Disease Surveillance System, the Department of Livestock Development (DLD), were used. Briefly, in Thailand, FMD surveillance includes clinical inspection and laboratory diagnosis when an outbreak is reported (passive) or as a result of active surveillance designed at the national level. The FMD outbreak records included (a) outbreak onset date (based on the appearance of first clinical signs), (b) the number of FMD outbreak episodes, and (c) location of the outbreaks (subdistrict, district, and province). Data were recorded in a spreadsheet format (Microsoft Excel), and then the data were exported to R statistical software version 3.6.3 (29) for data managements and analyses. Notably, the northern region was selected because this area had a high number of outbreak episodes, as well as the availability of the outbreak data. The study area consisted of 769 subdistricts within eight provinces in northern Thailand.

For this study, a case was defined as a subdistrict with one or more monthly “FMD-outbreak episodes,” between 2015 and 2017, based on livestock authorities' official records of farms with the presence of animals with clinical signs. To confirm disease and serotype, blood or tissue samples were collected by veterinary authorities from a subset of the animals with FMD-compatible clinical signs in a random subset of farms within each subdistrict in the region. Samples were processed at the Regional Reference Laboratory for Foot and Mouth Disease in Southeast Asia, DLD using reverse transcriptase–polymerase chain reaction and liquid phase–blocking enzyme-linked immunosorbent assay following the procedures described previously (30–32). The serotypes identified in the samples at the laboratory included serotypes O and A, and there was a group of samples in which the identification was unsuccessful. In this article, serotype O, serotype A, and the unidentified serotypes were grouped and referred to as overall serotype.

Because of differences in districts in time to respond or resources involved in outbreak investigations, the number of farms and the geographical extent of the “FMD-outbreak episodes” vary, limiting the ability to define an outbreak with more granular scale (e.g., effective reproductive number between farms).

### Characteristics of FMD Outbreak Episodes

The epidemic curve of FMD outbreak episodes of FMDV serotype O, serotype A, and the unidentified serotypes was created using R statistical software version 3.6.3 (29) with EpiCurve (33) and ggplot2 (34) packages. Furthermore, mapping of FMD outbreaks was constructed with Quantum Geographic Information System software version 3.12 (35) utilizing the WGS 1984 datum.

### Estimation of the Subdistrict Reproductive Number

We used the epidemic doubling time method to estimate *R*_*sd*_. This technique was described in detail in a previous report (25), but briefly, in the early stage of an epidemic, effective contact with an infectious individual will result in a certain rate of new infections when the population is completely susceptible. Based on this parameter, the number of secondary cases will increase dramatically, assuming the time in which the number of outbreaks doubles (i.e., time to doubling) remains constant. The linear relation between these two parameters has been reported as follows:


$$\begin{array}{l} R_{sd}=1+\left(\frac{D}{Td}\right)\;*\;\log2 \end{array}$$

where *D* is the duration of the infectiousness of an outbreak, assumed as 7 days as reported previously (36), and *T*_*d*_ is the time in which the number of outbreaks duplicates (23). We estimated *T*_*d*_ as follows:


$$\begin{array}{l} Td=\left(t2-t1\right)\;*\;\frac{\log\left(2\right)}{\log\left(q2/q1\right)} \end{array}$$

where *q* is the subdistricts affected with FMD (quantities) at time 2 (*t*_2_) and time 1 (*t*_1_) (37).

Epidemic curve was built by month, and the average time for the number of outbreaks to double (*T*_*d*_) for any conceivable combination was calculated. We assumed an infectious period of 1 month as the information was provided aggregated on a monthly scale. Statistical differences among *R*_*sd*_ by period of study were assessed with Kruskal–Wallis test. A statistical significance was concluded, if *p*-value that corresponded to the analysis was <0.05. Analyses were performed using R statistical software version 3.6.3 (29).

## Results

### Characteristics of FMD Outbreak Episodes in Northern Thailand

**Figure 1:**
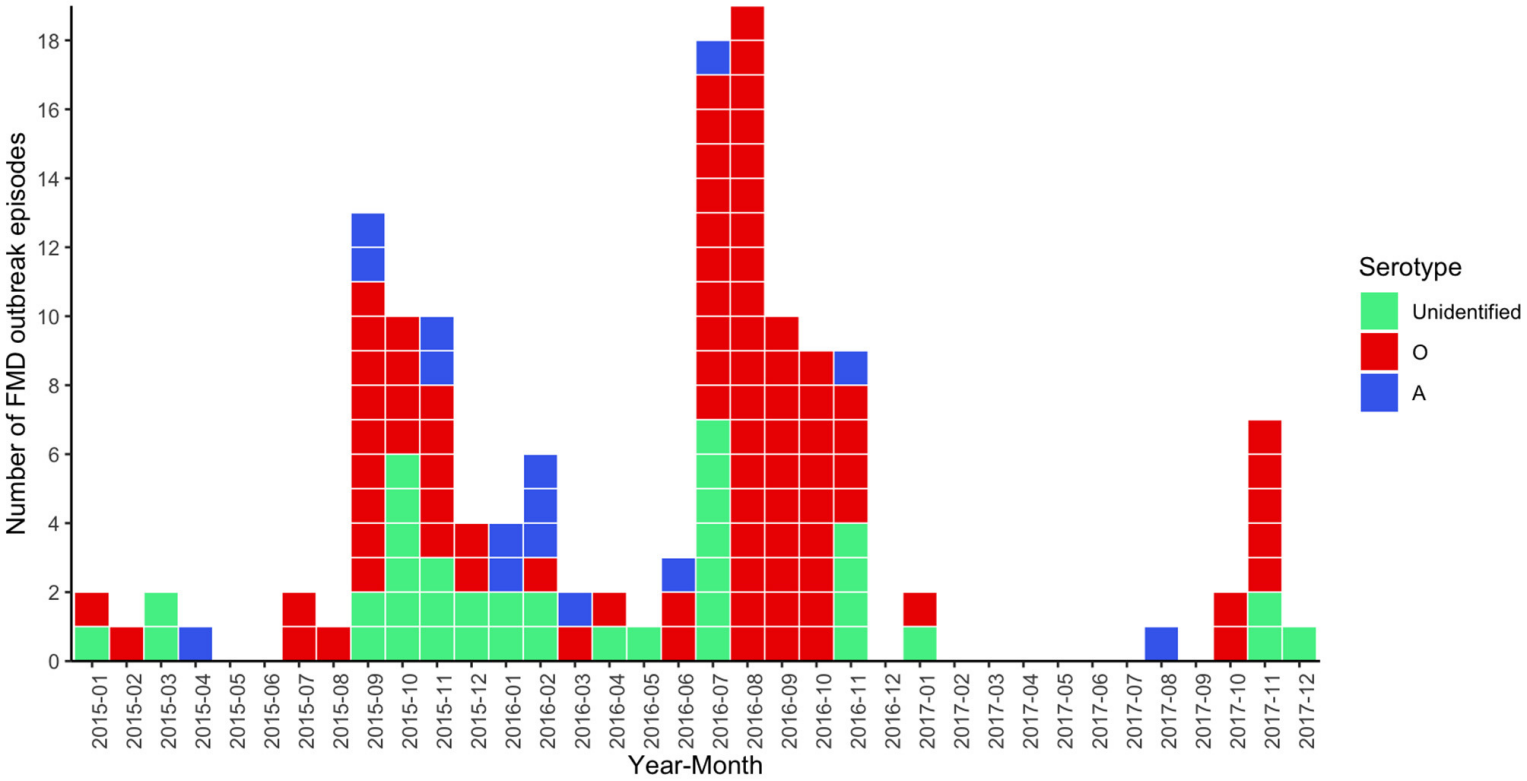
Epidemic curve for FMD outbreak episodes caused by FMDV serotype O (red, *n* = 90), FMDV serotype A (blue, *n* = 15), and unidentified serotype (green, *n* = 37) in cattle in northern Thailand, 2015–2017.

**Figure 2:**
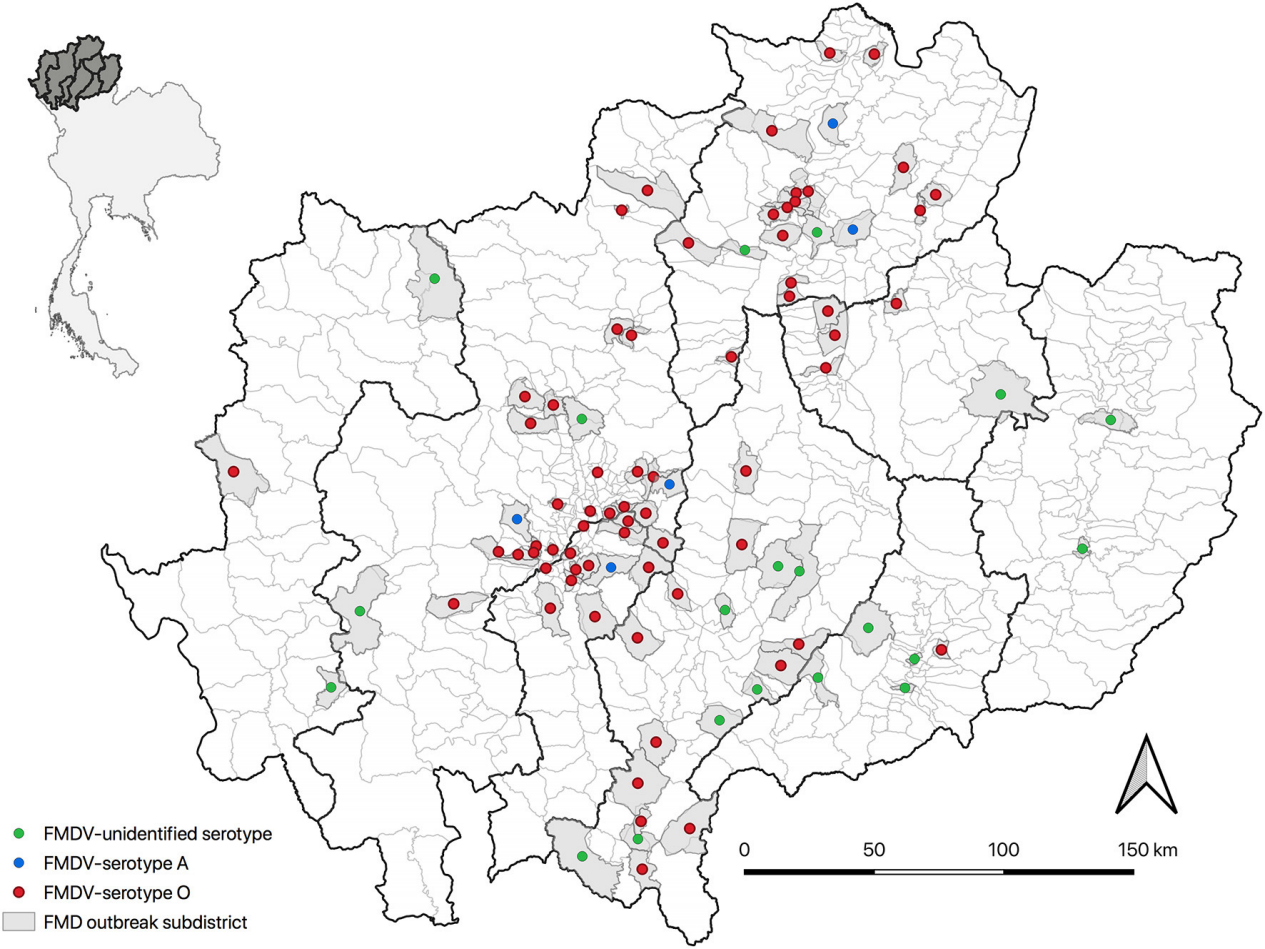
The spatial distribution of FMD outbreak episodes (*n* = 142) in cattle in northern Thailand (top left: gray color shade represents study provinces) from 2015 to 2017 by serotype in affected subdistricts (right).

Overall, there were 142 outbreak episodes of FMDV in cattle, and 90 (63.38%) of those were identified as serotype O between 2015 and 2017. Figure 1 illustrates three periods with a high number of FMD outbreaks at the subdistrict level, including the first in September 2015, the second in August 2016, and the third in November 2017. The means (and ranges) of subdistrict FMDV overall serotype and FMDV serotype O incidence in the region were 0.006 (0–0.028) and 0.003 (0–0.026), respectively. FMD episodes were observed in 12.22% (n = 94/769) of the subdistricts under study and were clustered in the center and north of the region of northern Thailand (Figure 2).

### Subdistrict Effective Reproductive Number (R_sd_)

The estimated *R*_*sd*_ using the epidemic doubling time for FMDV overall serotype, and FMDV serotype O outbreak episodes in cattle in northern Thailand for 2015–2017 remained constant over the years (Kruskal–Wallis *p* > 0.05), ranging from 1.02 to 1.06 for overall serotypes and 1.04 to 1.07 for serotype O, among the different periods studied (Table 1). The estimated average doubling times in cattle were 4.67 and 2.80 months, for the overall and serotype O outbreaks, respectively.

**Table 1:** Doubling time and subdistrict effective reproductive number (*R*_*sd*_) for FMD outbreaks by serotype in cattle in northern Thailand between 2015 and 2017.

**Date**	**Serotype**	***t*_2_ −*t*_1_**	** $\frac{q2}{q1}$ **	***T*_*d*_**	***R*_*sd*_**
Jan–Sep 2015	Overall	10	5.33	4.14	1.04
Feb–Aug 2016	Overall	13	3.50	7.19	1.02
Oct–Nov 2017	Overall	5	3.67	2.67	1.06
Jan–Sep 2015	O	7	10	2.11	1.07
Feb–Aug 2016	O	18	26	3.83	1.04
Oct–Nov 2017	O	3	2.33	2.45	1.06

*Overall, FMDV serotype O, serotype A and unidentified serotype; O, FMDV serotype O; t_1_, started month; t_2_, peak month; q_1_, number of subdistricts affected with FMD at started month; q_2_, number of subdistrict affected with FMD at peak month; T_d_, time in which the number of outbreaks duplicates; R_sd_, subdistrict effective reproductive number*.

## Discussion

To the best of our knowledge, this is the first study estimating FMD transmission parameters from field outbreaks in the small-scale cattle production system in northern Thailand. The findings from this study provide a better understanding of the dynamics of FMD disease transmission among cattle farms across subdistricts during the epidemic.

Results showed a low incidence of FMD outbreak episodes at the subdistrict level and many districts in which there was a lack of outbreaks recorded (81.5%). Low incidence, together with the observed clustering of similar serotypes observed (Figure 2), suggests a limited scale of the epidemic (19), which may be attributed to few contacts among cattle farmers across regions as a result of low animal density in the region and the small scale of farming (<30 animals) (38). Furthermore, this can be also the impact of control measures implemented by livestock authorities within those affected subdistricts, which restricted vehicle movements from the outbreak area to other areas (39). Notably, the results need to be interpreted with caution because the number of animals and farms involved in each outbreak episode could not be determined.

We determined *R*_*sd*_ using the time doubling method as the selection method for typical field situations in which information is incomplete or unavailable for tracing disease (25). Furthermore, as we determined *R*_*sd*_, there is a limitation to compare disease spread with other studies globally, which estimated transmission at the individual or herd level (18, 40, 41) or those estimating transmission in a completely susceptible population (*R*_0_) (19, 21, 42). Still, our study represents an estimate based on field conditions and outbreaks—recording limitations that can resemble other countries with similar constraints.

Despite the fact that the incidence of FMD outbreak episodes at the subdistrict level was low, the *R*_*sd*_ estimated in this study was higher than one, and the *R*_*sd*_ values are consistent with endemicity of FMD. Furthermore, the length of time in which cases were continuously observed (e.g., 9 months from January to September 2015) is consistent with an endemic setting. In addition, the low variability among estimates over time might be reflecting the impact of animal movement bans, FMD ring vaccination, and active serological monitoring (39). Although measures are in place, such as animal movement control and vaccination, it might not be optimal to eradicate the disease or fully control it; however, they might be sufficient to avoid further spread beyond the clustered area affected. An illegal animal movement may have occurred, although the animal movement measures were implemented. Moreover, control based on ring vaccination is performed for all cattle farms in the range of 20–50 km from the center of the outbreak area. However, it requires ~21 days to increase the immunity for FMD for vaccinated cattle (43). As the outbreak is an emergency, the selection of the type of vaccine strains (e.g., monovalent and trivalent) used for vaccination is based on the decision of the authority. Thus, immune responses may differ based on the type of vaccine used. Moreover, the routine vaccination is performed two to four times per year, but these practices are carried out at different months for each district; thus, if cattle are properly immunized in the different subdistricts, they will have different immunity levels, which can affect the susceptibility of disease at the time of a new incursion. A previous study also mentioned that the presence of FMDV serotypes O and A in 2016 nationwide poses difficulty in preventing and controlling the disease (30). In some areas, the ratio of antibody titer against the heterologous field strain and antibody titer against the homologous vaccine strain or *r* value (44) is not high, indicating that a moderate-level matching is observed between FMD vaccine strains and FMD outbreak (30). We suggested that FMD monitoring and control strategies should be strengthened to mitigate FMD outbreaks. The development of a smartphone application, for example, to report suspected FMD cases, which is important for rapid outbreak responses, may improve FMD outbreak notification system by shortening the time between finding the suspected cases and FMD case verification by authorities (45). Also, continuous monitoring of vaccine matching the antigenic characteristic of FMDV causing the outbreaks (46, 47) may lead to the control of FMD in Thailand where routine vaccination is performed (30).

It is important to note that our case definition was based mainly on clinical signs, which can impose some misclassification bias based on experience and training of personnel. However, Thailand has been FMD-endemic for more than 60 years (10), and standardized training and field experience of the official veterinarians are in place, increasing the accuracy of the clinical diagnosis and concordance among veterinarians. Furthermore, farmers in FMD-endemic areas have varying degrees of success in recognizing the FMD, as this skill is dependent on existing experience and understanding of cattle diseases. For example, dairy farmers who are members of dairy cooperatives are more likely than smallholder farmers who own backyard beef cattle to comprehend FMD as they have more group meetings to update animal health status according to cooperative policies.

The value of *R*_*sd*_ calculated in this study represents the effective reproductive number from the subdistrict unit found in the intervened population. Our results showed little difference (<0.03) in estimates when analyzing the most frequent serotype (i.e., O) compared to all FMD serotypes. This can indicate that serotype O is the most prevalent (63.38%), and disease transmission is mainly driven by its characteristics. As we intended to estimate *R*_*sd*_ for each FMD serotype circulation in Thailand (12), the lack of records impaired the analysis. Although not optimal, values were obtained from all FMD outbreaks to increase the understanding of the epidemiological situation in the country and are valuable evidence to show endemicity among subdistricts studied. As mentioned previously, knowing the FMD serotype endemicity can support livestock authorities in developing more effective vaccination programs (30).

In Thailand, recording FMD outbreak data among cattle herds as the origin of each outbreak is usually a limitation. Although it is feasible to calculate *R*_0_ during a newer epidemic of an infectious disease that is transmitted across a fully susceptible population, the data collection systems in Thailand are not developed enough to identify the early stages of an epidemic when *R*_0_ is most reliably determined. The reporting times are often inconsistent, and the collection of reliable contact tracing data is usually unfeasible. Although in this study we were able to estimate and demonstrate the value of the *R*_*sd*_, there is a need to understand FMDV transmissibility within and between farms to fully control the disease in Thailand. Hence, it is important to collect suitable data for this end with a standardized data collection protocol at the field level.

Given that FMD is a reportable disease in Thailand, we presume that all outbreaks in northern Thailand have been reported and officially confirmed during the study period. Yet, some FMD cases might not be reported during epidemics (27), as other factors include lack of attention to detect FMD symptoms of farmers, lack of some reliable and accurate sample collection for laboratory examinations, or incursions located in remote areas.

Because of reporting data limitations, the epidemiological unit of interest was subdistrict level. Livestock databases still have potential in disease simulations; however, the challenge is to build comprehensible models that can be related to disease surveillance data collected in Thailand (48), which lacks the demographic information of villages or the cattle farms in the country. We recommend that livestock authorities expand active surveillance activities, such as regular farm visits and more interaction with smallholder farmers, as well as to strengthen the passive surveillance system, such as encouraging cattle farmers to report FMD outbreaks as soon as possible.

In conclusion, with the quantification of the epidemiological parameter *R*_*sd*_ of the FMDV (overall and serotype O), this study indicates that the FMD incursions in cattle in northern Thailand are likely to remain endemic even with current control interventions. This result highlights the need for improvement in the granularity of surveillance data collection and the reevaluation of control strategies in the northern region to reach control of the disease.

## Data Availability Statement

The datasets generated for this study will not be made publicly available as the data has been provided by the authority of the Department of Livestock Development, Ministry of Agriculture and Cooperatives, Thailand. Requests to access these datasets should be directed to info@dld.go.th.

## Author Contributions

VP, AP, and CP-R: conceived the study. OA, CP-R, and VP: analyzed the data and wrote the main manuscript text. AP supervised the study. OA, VP, AP, and CP-R: contributed to the interpretation of the result. All authors contributed to the article and approved the submitted version.

## Funding

This work was funded by Chiang Mai University (Grant numbers: R000026522 and R000026062). The funder had no role in the study design, data analysis, decision to publish, or manuscript preparation.

## Conflict of Interest

The authors declare that the research was conducted in the absence of any commercial or financial relationships that could be construed as a potential conflict of interest.

## Publisher's Note

All claims expressed in this article are solely those of the authors and do not necessarily represent those of their affiliated organizations, or those of the publisher, the editors and the reviewers. Any product that may be evaluated in this article, or claim that may be made by its manufacturer, is not guaranteed or endorsed by the publisher.
